# Supplementary material for: The prevalence of molecular markers of resistance to sulfadoxine-pyrimethamine among pregnant women at first antenatal clinic attendance and delivery in the forest-savannah area of Ghana
Source: PLoS One. 2022 Aug 8;17(8):e0271489. doi: 10.1371/journal.pone.0271489 (PMC9359546; doi:10.1371/journal.pone.0271489)
Supplement: S1 Table — (DOCX) [file pone.0271489.s001.docx]

| Characteristics | Included, n (%)  (N=255) | Not included,  n (%) (N=62) |
| --- | --- | --- |
| Maternal Age (years) |  |  |
| ≤24 | 124 ( 48.6) | 28 (45.2) |
| 25 – 34 | 98 (38.4) | 25 (40.3) |
| ≥35 | 24 (9.4) | 5 (8.1) |
| Missing | 9 (3.5) | 4 (6.5) |
| Highest educational level |  |  |
| None | 86 (33.7) | 19 (30.7) |
| Primary school | 51 (20.0) | 8 (12.9) |
| Junior High/ Middle School | 74 (29.0) | 26 (41.9) |
| Secondary School or higher | 41 (16.1) | 6 (9.7) |
| Missing | 3 (1.2) | 3 (4.8) |
| Marital status |  |  |
| Married/married before | 161 (63.1) | 42 (67.7) |
| Living together with a man/unmarried | 39 (15.3) | 7 (11.3) |
| Single, unmarried | 52 (20.4) | 10 (16.1) |
| Missing | 3 (1.2) | 3 (4.8) |
| Malaria parasite density (parasites/µL of blood) |  |  |
| Range | 20 - 46500 | 25 - 6443 |
| Geometric mean | 522 | 312 |
| Temperature >37.5 ^o^C |  |  |
| Yes | 1 (0.4) | 0 (0.0) |
| No | 241 (94.5) | 55 (88.8) |
| Missing | 13 (5.1) | 7 (11.3) |
