# Supplementary material for: The prevalence of molecular markers of resistance to sulfadoxine-pyrimethamine among pregnant women at first antenatal clinic attendance and delivery in the forest-savannah area of Ghana
Source: PLoS One. 2022 Aug 8;17(8):e0271489. doi: 10.1371/journal.pone.0271489 (PMC9359546; doi:10.1371/journal.pone.0271489)
Supplement: Attachment — Submitted filename: Review report 2.docx [file pone.0271489.s002.docx]

**Review report of manuscript titled "** **"The prevalence of molecular markers of resistance to Sulphadoxine-pyrimethamine among pregnant women at first antenatal clinic attendance and delivery in the forest-savannah area of Ghana.”(PONE**-D-**22-03757**) **by Kwame, D. et al.**

The present manuscript evaluates the prevalence of the marker mutations associated with antimalarial drugs (Sulphadoxine and pyrimethamine) resistance among pregnant Women at ANC and at the time of delivery in Ghana. The study has been designed well and appropriate statistical tools have been used to analyse the data. I congratulate the authors for providing scientific evidence for monitoring the emergence and spread of drug resistance in malaria endemic country of Ghana.

**Major comments:**

1. How did you calculate the sample size?

2. Further, why there is variation in total number of samples analysed for different mutations at ANC and at the time of delivery?

3. The average doses compliance was 2.9 doses. It is a known fact that poor doses compliance promotes drug resistance. Kindly discuss the situation of drug resistance in light of doses compliance in discussion section.

**Minor comments:**

1. Page no. 11, line no 207, the value of percentage should be 83% (157/189) in place of “81%.(157/189)”

2. Page no. 11, line no 213, the value of percentage should be 76.2% (138/181) in place of “72.6 % (138.181)”

3. Page no. 11, line no 215, the value of percentage should be 72% (18/25) in place of “76.2 % (18/25)”

Decision : Publish after modification.
